# Spontaneous regression of central nervous system posttransplant lymphoproliferative disease: A case report

**DOI:** 10.1097/MD.0000000000024713

**Published:** 2021-02-12

**Authors:** Rupan Gao, Yue Zhang, Gong Chen, Abhijeet Kumar Bhekharee, Zunguo Du, Shuguang Chu

**Affiliations:** a Department of Hematology, Zhongshan Hospital; b Department of Neurology; c Department of Neurosurgery, Huashan Hospital; d Shanghai Medical College, Fudan University; e Department of Pathology, Huashan Hospital, Fudan University; f Department of Radiology, Shanghai East Hospital, Tongji University School of Medicine, Shanghai, China.

**Keywords:** epstein-barr virus, polymorphic posttransplant lymphoproliferative disease, spontaneous remission

## Abstract

**Rationale:**

Primary central nervous system (CNS) posttransplant lymphoproliferative disease (PTLD) is a very rare entity. Patients may respond to reduction of immunosuppression or other therapies, but the prognosis is still pessimistic.

**Patient concerns:**

Herein, we report a 40-year-old female with a history of renal transplantation developed brain masses 4 years ago. Although brain biopsy was performed, PTLD was underdiagnosed then. No relevant treatment was administered. However, the lesions resolved spontaneously. After 4 years, new lesion appeared in a different brain region.

**Diagnoses:**

The history of renal transplantation raised the suspicion of PTLD. Reexamination of previous brain sections confirmed the diagnosis of polymorphic PTLD (P-PTLD). A second biopsy of the new lesion also demonstrated P-PTLD.

**Interventions:**

She was referred to hematology department to receive rituximab.

**Outcomes:**

After 4 rounds of treatment, the lesion resolved satisfactorily.

**Lessons:**

This case demonstrates the natural history of primary CNS P-PTLD. Although self-remission and recurrence is possible, aggressive measures should be taken to this condition.

## 1 Introduction

Posttransplant lymphoproliferative disease (PTLD) is a group of lymphoproliferative diseases in the recipient of solid organ or stem cell allograft. Median time from transplant to PTLD is around 4.5 years.^[1]^ This condition is highly associated with Epstein-Barr virus (EBV) infection.^[2]^ EBV infection is present in 60% to 70% of cases.^[3]^ Primary central nervous system (CNS) PTLD is an extremely rare entity. The annual incidence of PTLD is 1% to 10% and primary CNS PTLD only account for 5% to 15% of all PTLD cases.^[4]^ Pathologically, most CNS PTLDs are monomorphic, indicating poor prognosis.^[1]^ Treatment such as reduction of immunosuppression (RI), rituximab, chemotherapy, surgery, radiation, immunotherapy, and antiviral therapy may show some effects,^[5]^ but long-term prognosis is far from optimistic. We report a case of CNS polymorphic PTLD (P-PTLD) which was underdiagnosed 4 years ago achieved spontaneous remission then. After being stable for 4 years, new lesion appeared in a different brain region. Brain biopsy still suggested P-PTLD. The lesion resolved after 4 rounds of rituximab treatment.

## 2 Case presentation

**Figure 1:**
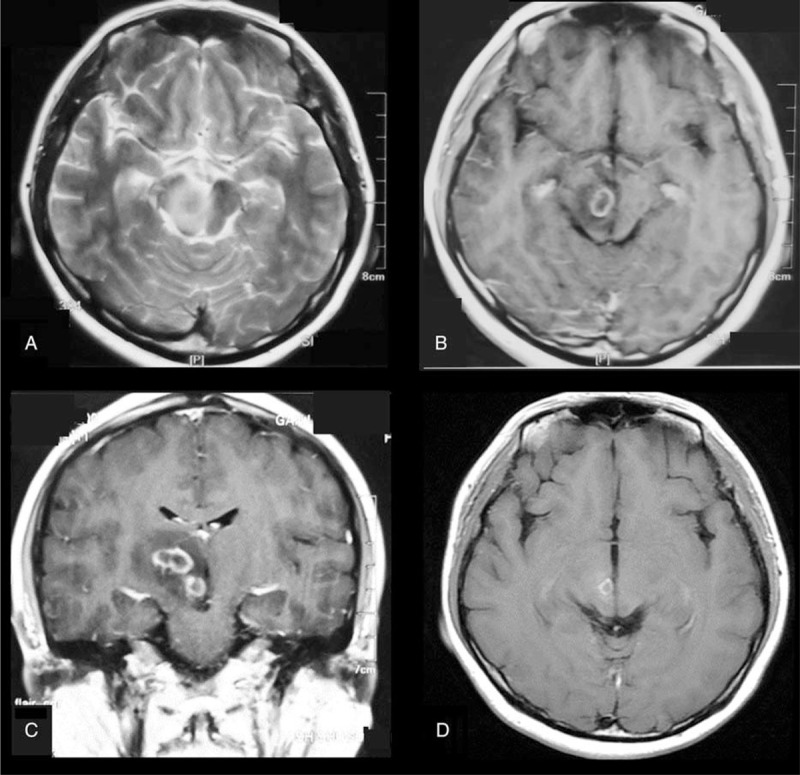
MRI findings in 2016 Axial T2WI (A) and post contrast T1WI (B) showed a rim-enhanced lesion in the right midbrain surrounded by vasogenic edema. Sagittal post contrast T1WI (C) revealed 2 masses in the right midbrain and thalami. Post contrast T1WI 1 month later (D) showed resolution of the lesion.

A 40-year-old female presented in the clinic with 1-month-long progressive right-sided visual field defect. In the past history, she received kidney transplant 17 years ago because of renal failure caused by IgA nephropathy. After surgery, 1.25 g mycophenolate and 4 mg methylprednisolone were used daily to prevent allograft rejection. Four years ago, she developed double vision and left-sided weakness. Brain MRI revealed right thalamus and midbrain rim-enhanced lesions, mimicking glioblastoma multiforme (Fig. 1 A-C). However, stereotactic brain biopsy revealed lymphoid hyperplasia rather than glioma. After the surgery, she did not adjust the dose of mycophenolate and methylprednisolone nor receive any treatment. However, the lesions dramatically resolved 1 month later (Fig. 1 D). During the past 4 years, sequential brain MRIs did not reveal new lesions.

**Figure 2:**
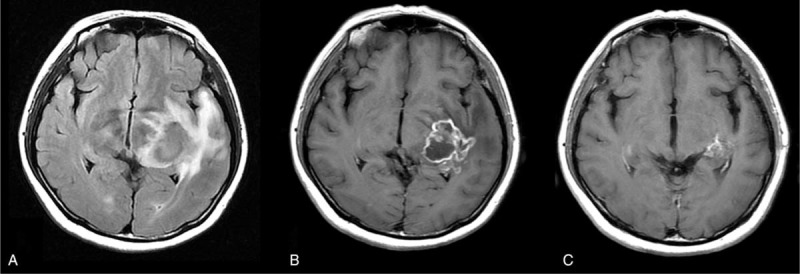
MRI findings in 2020 FLAIR (A) showed a hypointense mass in the left temporal lobe with severe vasogenic edema. Post contrast T1WI (B) showed irregular rim-enhancement. The lesion regressed significantly after rituximab treatment (C).

**Figure 3:**
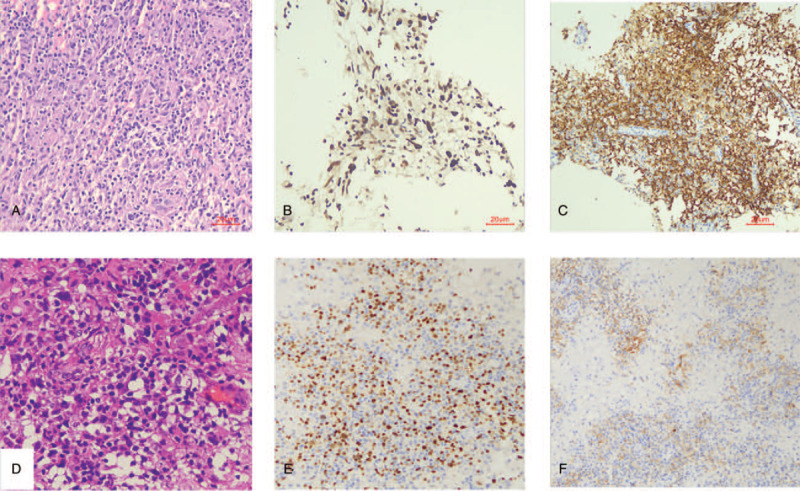
Pathology findings HE staining (origin × 400) of right thalami lesion in 2016 (A) showed abundant polymorphous lymphocytes infiltration. EBER (B) and CD20 stains (C) were positive. HE staining (origin × 400) of left temporal lesion in 2020 showed polymorphous lymphocytes infiltration (D). EBER (E) and CD20 (F) stains were also positive.

On admission, neurological examinations found right visual field defect. Leukocytes count was 5.63 × 10^9/L. Erythrocytes count was 3.59 × 10^12/L. Hemoglobin was 113 g/L. Platelet count was 124 × 10^9/L. Liver function and renal functions were normal. HIV test was negative. Blood EBV DNA was 2.86E+04 copies/mL (reference range:< 5.0E+02). EB serostatus was as follows: anti EBNA IgG 241U/mL (<5U/mL), anti-VCA IgG 620U/mL (<20U/mL), anti-EBEA IgG 82U/mL (<10U/mL), anti-VCA IgM negative. Lumbar puncture revealed opening pressure of 165mmH_2_O. Cerebrospinal fluid (CSF) leukocyte count was 12 × 10^6/L. CSF protein level was 0.98 g/L (reference range:0.15–0.45). Next-generation sequencing of CSF detected 211 sequence reads of EBV. Brain MRI demonstrated a rim-enhanced mass in the left temporal lobe (Fig. 2, A). Given long-term use of anti-rejection drugs and positive EBV DNA in CSF, PTLD was suspected. Previous brain sections were reexamined. The lymphocytes were polymorphous and stained diffusely and positively with CD20, CD30, and EBER but negatively with CD2, CD4, and CD8, consistent with P-PTLD (Fig. 3). Ki67 was 50%. B-cell gene rearrangement was positive. For fear of the possibility of evolution of P-PTLD to lymphoma, a second brain biopsy was performed. Pathology still suggested P-PTLD (Fig. 3). She was referred to hematology department and received 500 mg rituximab injection every month for 4 months. The dose of mycophenolate was temporarily not modified for fear of graft rejection. Her vision field gradually recovered and brain MRI showed significant improvement (Fig. 2).

## 3 Discussion and conclusion

PTLD is a rare lymphoproliferative disease in patients who received organ or bone marrow transplant. Primary CNS PTLD is only account for 5% to 15% of all PTLD cases.^[4]^ This condition is more likely to be associated with kidney transplant^[1]^ and application of mycophenolate mofetil.^[1,2]^ The major tool leading to the diagnosis of CNS PTLD is brain biopsy. Pathological findings of PTLDs are subdivided into early lesions, P-PTLD, monomorphic PTLD, and Hodgkin lymphoma (HL)-like variants,^[6]^ while most CNS PTLDs are monomorphic, forecasting poor prognosis.^[1]^ Radiological profile is unspecific. MRI may reveal multiple masses, central necrosis, hemorrhage, surrounding vasogenic edema, and ring enhancement. But glioma, metastasis, toxoplasmosis, abscess, and tuberculosis may share similar appearance. MRI is also unlikely to tell P-PTLD from other pathological subtypes.^[7–10]^ If the past history of kidney transplantation was not paid attention to, the diagnosis of CNS PTLD would be challenging.

The basic treatment of PTLD is RI which alone is occasionally effective in early lesions or P-PTLD.^[10,11]^ When RI shows no effect, rituximab, chemotherapy, surgery, radiation, immunotherapy, and antiviral therapy should be considered.^[5]^ For CNS PTLDs, monomorphic PTLD counts for 83% of CNS PTLD^[1]^ and RI alone seems ineffective.^[10]^ There are only a few reports of CNS P-PTLD.^[7–10]^ Whether treatment specific for P-PTLD is needed is not known. Kesari NK et al reported a case which was treated with RI, dexamethasone, rituximab and sirolimus.^[7]^ Morris J, et al reported a patient treated with RI, rituximab and cranial radiation.^[8]^ Arita H, et al presented two cases of P-PTLD treated with RI plus steroids and surgery plus irradiation separately.^[9]^ Velvet AJJ, et al reported a case treated with RI, craniotomy, radiotherapy and rituximab.^[10]^ The outcomes of these cases are optimistic, but due to the rarity, there is no consensus of optimal treatment. What made our case so special is that the patient did not received RI and the lesion resolved spontaneously. Her condition even remained stable for 4 years. Spontaneous remission occasionally occurred in lymphoproliferative diseases and even in lymphomas. Blokx, W. A. reported a case of posttransplant CNS B-cell lymphoma experienced spontaneous regression initially but the tumor spread all over the brain 2 months later.^[12]^ Snijder J reviewed 17 cases of diffuse large cell lymphoma which achieved spontaneous remission. EBV was detected in 36% of this group, so the authors assume the mechanism behind self-remission is significant antigenicity conferred by EBV viral sequences incorporated within tumor genome.^[13]^

In conclusion, we present a case of CNS P-PTLD which resolved spontaneously 4 years ago and relapsed 4 years later. History of organ transplantation or stem cell allograft, evidence of EBV infection, and characteristic MRI findings are helpful hints, but the diagnosis should be established on histopathology. Although it is possible that waxing and waning is the natural progress of CNS P-PTLD, this does not justify not taking aggressive measures to this condition.

## Author contributions

G.R.P: initial draft manuscript preparation, analysis the radiologic data. Z.Y.: initial draft manuscript preparation, patient management. C.G: patient management, analysis the radiologic data. D.Z.G.: literature review, providing pathological images. B.A.K. critical review of the manuscript. C.S.G.: critical review of the manuscript, final approval of the version to be submitted. All authors have read and approved the manuscript. G.R.P. and Z.Y. contribute equally to the work. Written consent to publish was obtained from the patient.

**Conceptualization:** Shuguang Chu.

**Data curation:** Gong Chen.

**Formal analysis:** Zunguo Du.

**Writing – original draft:** Rupan Gao, Yue Zhang.

**Writing – review & editing:** Abhijeet Kumar Bhekharee.

## Abbreviations

Abbreviations: CNS = Central nervous system, CSF = Cerebrospinal fluid, EBV = Epstein-Barr virus, P-PTLD = polymorphic posttransplant lymphoproliferative disease, PTLD = Posttransplant lymphoproliferative disease, RI = reduction of immunosuppression.
